# Dichorionic Diamniotic Twin Pregnancy Discordant for Bladder Exstrophy

**DOI:** 10.1155/2009/186483

**Published:** 2009-09-13

**Authors:** William Tu, Jane Chueh, William Kennedy

**Affiliations:** ^1^Department of Urology, Stanford University, 300 Pasteur Drive, Stanford, CA 94305, USA; ^2^Department of Obstetrics and Gynecology, Stanford University, 300 Pasteur Drive, Stanford, CA 94305, USA

## Abstract

A 38 year-old woman presented with a dichorionic diamniotic twin pregnancy at gestational age of 32 weeks concerning for an abdominal wall mass in one of the twins. Initial ultrasound evaluation was suspicious for an omphalocele, but the affected twin was found to have bladder exstrophy at birth. This illustrates the difficulties of accurate prenatal diagnosis of bladder exstrophy in a twin pregnancy at a late gestation.

## 1. Introduction

Bladder exstrophy is a rare congenital malformation which occurs as a result of incomplete closure of the lower anterior abdominal wall. The incidence ranges from 1 : 10, 000 to 50,000 live births with a male to female ratio of 2 : 1 [1, 2]. The embryologic defect is failed mesenchymal cell migration between the cloaca and abdominal wall ectoderm. This leads to an absence of anterior abdominal connective tissue and muscles. 

 Prenatal diagnosis involves differentiating this defect from other common anterior abdominal wall defects including omphalocele and gastroschisis. Ultrasonography has been the standard diagnostic modality but may be limited in twin pregnancies and late gestational presentations. Proper identification is important for prenatal counseling and preparing surgical teams for expectant management. We describe the sonographic findings and management in an unusual case of bladder exstrophy.

## 2. Case Presentation

**Figure 1:**
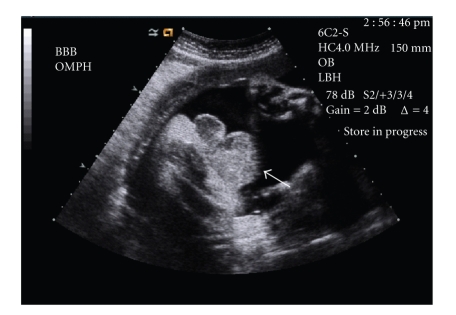
Fetal ultrasound of twin B illustrating anterior wall defect (arrow).

A 38-year-old, gravida 8, para 1-0-6-1, woman presented with a dichorionic diamniotic twin intrauterine pregnancy with discordant growth. Twin A appeared normal but twin B was 37% smaller. Amniocentesis at 18 weeks gestation showed both twins with karyotype 46 XY. Fetal echocardiogram at 28 weeks on twin B was concerning for possible ventriculoseptal defect and coarctation of the aorta. Ultrasound at a late gestational age of 32 weeks on twin B showed normal amniotic fluid and renal development. However, a filling urinary bladder was not clearly visualized in twin B compared to twin A. A 2-3 cm diameter lower abdominal mass and extracorporeal liver with an entering vessel suspicious for omphalocele was seen on twin B (Figure 1). 

**Figure 2:**
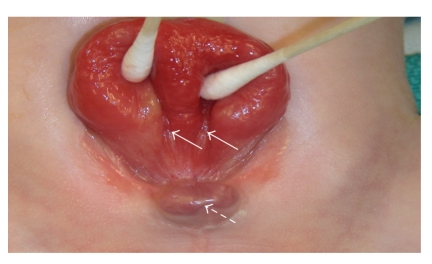
Closeup of bladder plate indicating location of ureteral orifices (solid arrows) and phallus with epispadias (dashed arrow).

The patient was referred to pediatric surgery for counseling on abdominal wall defects, specifically omphalocele and gastroschisis. At 35 6/7 weeks, the patient underwent cesarean section delivery. Examination of newborn B showed a low set umbilicus, pubic bone diastasis, and epispadias. The anterior abdominal wall mass was infraumbilical with a large, everted mucosalized surface and identifiable ureteral orificies consistent with bladder exstrophy (Figure 2). 

At birth, the pediatric urology service was consulted for management of twin B. An echocardiogram showed a minor patent ductus arteriosus without a ventriculoseptal defect or coarctation of the aorta. On day one of life, twin B, weighing 1.8 kg, was taken for a classic staged exstrophy-epispadias repair first with primary bladder closure. The pubic symphysis was rigid and fixed with a pubic bone diastasis of approximately 3 cm. Osteotomies were not performed due to the risk of increased blood loss and additional operative time. 

**Figure 3:**
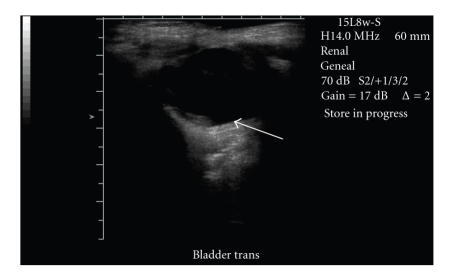
Ultrasound of pelvis of twin B at 2-month followup showing intact abdominal wall with full bladder (arrow).

 Twin B had an uneventful recovery from the first stage of the bladder exstrophy repair and was discharged home on postoperative day 38. At his 2 month followup clinic visit, his parents reported some nights with a dry diaper, reflective of good bladder capacity and hopeful urinary continence. Renal bladder ultrasound showed growing kidneys and a filled bladder (Figure 3).

## 3. Discussion

Congenital anterior abdominal wall defects are frequently diagnosed on prenatal ultrasound. Anatomic evaluation is limited in twin pregnancies by the space to identify distinct structures. Optimal imaging of these defects occurs at gestational age of earlier than 20 weeks. The differential diagnosis of anterior abdominal wall defects includes omphalocele, gastroschisis, bladder exstrophy, and cloacal exstrophy. Cloacal exstrophy is a combination of bladder exstrophy and omphalocele with other congenital anomalies. Unlike bladder or cloacal exstrophy, omphalocele and gastroschisis are associated with a filling and emptying bladder. A persistent urachal cyst may also be misinterpreted as a fluid filled bladder separate from the lower abdominal mass but would not show dynamic function [3]. Since fetal urine production begins at 8 weeks gestation, a functional bladder should be visualized by 15 weeks gestation [3]. In the absence of renal abnormalities or oligohydramnios, bladder exstrophy should be suspected. 

 Another technique to differentiate omphalocele from bladder exstrophy is color Doppler ultrasonography of the umbilical arteries in the axial plane. The umbilical arteries arise from the internal iliac artery and course around the bladder. Identification of the arteries running alongside an anterior abdominal wall mass is suggestive of bladder exstrophy [1, 3]. With an omphalocele, the arteries course inferior to the abdominal wall mass and an umbilical vein runs into the herniated liver associated with the mass. Assessment of the anatomic relationship between the umbilical arteries and anterior abdominal wall mass facilitates the proper diagnosis. In addition to ultrasound, magnetic resonance imaging can be used to identify an abdominal wall mass as the bladder based upon the course of the ureters. Fetal magnetic resonance imaging is not limited by the relative paucity of amniotic fluid during late gestation [4]. 

 Bladder exstrophy is commonly associated with epispadias and pubic bone diastasis. Prenatal counseling should address not only prognosis of urinary continence and renal function from bladder repair but also psychosexual outcomes from penile repair [5, 6]. When the pregnancy is continued to delivery, pediatric urology and pediatric orthopedics should be notified in advance to prepare for repair of the exstrophy-epispadias and pubic bone diastasis. The pubic symphysis is generally malleable from the influence of the maternal hormone relaxin after a vaginal delivery in the first 24 hours of life but the effect quickly diminishes by 48–72 hours of life. When the diastasis is less than 4 cm, then primary repair may be attempted without osteotomies [5, 7]. Since our patient had a premature cesarean section delivery, relaxin was not elevated in the circulation to have its effect so the pubic symphysis was rigid. 

 Our reported case was unique from the late presentation of the patient with a twin pregnancy and preterm delivery. When a fetal bladder is never clearly seen, suspicion for bladder exstrophy should be raised. Color doppler ultrasonography and fetal magnetic resonance imaging can be used to delineate the relationship of the umbilical artery and ureters to an anterior abdominal mass to differentiate bladder exstrophy from omphalocele or gastroschisis. The diagnosis of bladder exstrophy is important for prenatal counseling and early assemblage of the necessary surgical teams for treatment after delivery.
